# Diversity of attachment systems in heelwalkers (Mantophasmatodea) – highly specialized, but uniform

**DOI:** 10.1186/s12862-024-02319-x

**Published:** 2024-10-25

**Authors:** Thies H. Büscher, Stanislav N. Gorb, Monika J. B. Eberhard

**Affiliations:** 1 Department of Functional Morphology and Biomechanics, Kiel University, Am Botanischen Garten 1-9, 24118 Kiel, Germany; 2 Institute of Cell and Systems Biology of Animals, Department of Biology, University of Hamburg, Martin-Luther-King Platz 3, 20146 Hamburg, Germany

**Keywords:** Adhesion, Sexual dimorphism, Functional morphology, Locomotion

## Abstract

**Background:**

Heelwalkers possess a highly modified tarsal attachment system. All extant species lift the distalmost tarsomere permanently off the substrate and primarily use their euplantulae for locomotion. The combination of a smooth adhesive pad (arolium) on the pretarsus and fibrillary attachment pads on the euplantulae offers valuable insights for translational approaches, but its infra-order diversity remains unexplored.

**Results:**

We explored the morphology of the tarsal attachment apparatus of Mantophasmatodea based on a representative taxon sampling spanning a large fraction of species of this group and compared morphological differences in the specialized morphology of this system across species and sexes. Our scanning electron microscope investigation of the tarsi of 11 species (52% of all described extant species) revealed an overall very consistent ground pattern and almost no specific adaptations. There are only minor, but mostly clade-specific differences in the shape of the adhesive setae on the tarsal euplantulae and in the morphology and density of the acanthae on the pretarsal arolium. Both features differ primarily between Austrophasmatidae in comparison to the remaining Mantophasmatodea taxa.

**Conclusion:**

We conclude that the strong specialization of the mantophasmatodean tarsal attachment sufficiently copes with the diversity of substrates the insects are exposed to.

**Supplementary Information:**

The online version contains supplementary material available at 10.1186/s12862-024-02319-x.

## Introduction

Attachment devices for locomotion are considered key innovations during the evolution of insects playing a major role for their diversification [1–3]. They promoted diversification of habitats and lifestyles [4] and enable extant insects to move over various terrains [5]. The adaptation to different surfaces in various environments resulted in a plethora of different attachment devices on the tarsi and pretarsi of insects [1, 6, 7].

Heelwalkers (Mantophasmatodea) stand out among insects for various reasons. Their description in 2002 [8] as the latest described insect order caused considerable interest for insect systematics [6, 7, 9], and their characteristic tarsal architecture and functionality distinguish heelwalkers from all other insects [6, 7, 10]. These apterous predators are at best medium sized and primarily occur in southern parts of Africa [11, 12]. Compared to the tarsal morphology of other insects, the tarsi of Mantophasmatodea are highly specialized [6, 7]. One striking apomorphic trait is the eponymous modification of the 5th tarsomere, which is usually lifted off the substrate [10]. This tarsomere bears the pretarsus with two claws strongly reduced in size and an enlarged arolium [6, 7, 13]. For attachment during locomotion, heelwalkers employ only the attachment pads of the proximal tarsomeres (euplantulae), which are densely covered with elongated adhesive setae [6, 7]. The pretarsal arolium in contrast bears acanthae (smaller cuticular outgrowths) on a large fraction of its surface and a smooth area without any surface projections, which is brought into contact with the substrate for generation of adhesion [10]. While the adhesive force that can be generated by the arolia is remarkably high and allows the animals to attach to smooth substrates with one tarsus only (see [14]), these pads are mostly used in rare occasions, such as for attachment support in emergency situations [6], during feeding [10, 14] or copulation [10]. The general tarsal equipment was described for one species in detail [6, 7] and one study investigated the arolium of two further species [10]. These studies summarized the putative tarsal ground pattern for Mantophasmatodea to consist of five tarsomeres, of which the basal three are synsclerotic, but separated by distinct dorsal grooves [15] and a pretarsus, both equipped with the respective attachment pads [6, 7, 10]. Notably, the combination of hairy euplantulae and smooth arolia unites the two main principles of tarsal attachment devices in insects [2] on the same tarsus. There are various convergent occurrences of either hairy or smooth attachment pads in animals [3, 5], and only few cases of a combination of these principles within the same individual or species. Despite their morphological difference, both principles rely on maximization of the actual contact area for attachment and achieve this by flexible setae in hairy systems [2, 16] or by soft cuticle layering in smooth systems [17] aiding in adaption to the asperities of the substrates. Both are included in the specialized morphology of tarsi of Mantophasmatodea. This hybrid system is of interest to investigate complementary functions of both pad types for translational approaches [4]. Exploring its diversity within Mantophasmatodea aids in identification of adaptive modifications and limitations of this combination of attachment principles.

The tarsal attachment structures of some other polyneopteran insect groups vary considerably between species, e.g. in earwigs (Dermaptera; [18]) stone flies (Plecoptera; [19]) and stick and leaf insects (Phasmatodea; [20, 21]), resulting from ecological differences of the species [3, 5]. In contrast, in Zoraptera (angel insects) tarsal features are very uniform [22]. Zoraptera comprises only few species and it has been speculated that the absence of specialized tarsal attachment structures interfered with the diversification of this specific lineage [22]. Mantophasmatodea is as well represented by only a small number of species [23], but these possess remarkably complex tarsal attachment systems. As the morphology of the attachment systems of only few taxa is known in this group, we intend to shed light on the diversity of this character system within Mantophasmatodea.

We investigated the tarsal morphology of a broad taxon sampling across the major lineages of Mantophasmatodea. Ten species, six represented by both sexes, were examined using scanning electron microscopy (SEM). We compared the tarsal morphology of these species and incorporated the illustrations present in the literature [6, 7, 10] to analyze the diversity of the attachment system in this lineage based on 52% of all known extant species (eleven species). Our aim was to elucidate (i) the diversity of this specialized attachment system, (ii) potential adaptations at the species-level and (iii) potential sexual dimorphism in the attachment system.

## Materials and methods

### Specimens

We examined the tarsi of adult specimens of various mantophasmatodean species as listed in Table 1. All samples were preserved in 70% ethanol from previous studies [10, 23, 24]. The tarsal morphology of two further species was visualized in previous studies and included in this analysis. For one of these species, *Mantophasma zephyra*, additional micrographs not included in Beutel & Gorb [6, 7] were used to assess the features of concern for this study.

**Table 1:** Species used in the present study. *n* indicates the number of specimens examined in this study. * = information taken from the literature

Species	origin	sex examined (*n*)
Mantophasmatidae		
*Sclerophasma paresisense* Klass, Picker, Damgaard, van Noort & Tojo, 2003	captive bred, 2006;	male (1), female (1)
*Mantophasma zephyra* Zompro, Klass, Kristensen & Adis, 2002	Namibia, from Beutel & Gorb [6]	female (1)
*Mantophasma kudubergense* Zompro & Adis, 2006	Eberhard et al. [10]	male, female (*)
*Tyrannophasma/Praedatophasma* clade		
*Tyrannophasma gladiator* Zompro, 2003	captive bred, 2006;	male (1), female (1)
Austrophasmatidae		
*Hemilobophasma montaguense* Klass, Picker, Damgaard, van Noort & Tojo, 2003	Montagu, RSA, 2006/07	male (1)
*Austrophasma gansbaaiense* Klass, Picker, Damgaard, van Noort & Tojo, 2003	DeKelder, RSA, 2006/07	male (1), female (1)
*Austrophasma rawsonvillense* Klass, Picker, Damgaard, van Noort & Tojo, 2003	Rawsonville, RSA, 2006/07	female (2)
*Namaquaphasma ookiepense* Klass, Picker, Damgaard, van Noort & Tojo, 2003	Kamieskroon, RSA, 2006/07	female (1)
*Karoophasma biedouwense* Klass, Picker, Damgaard, van Noort & Tojo, 2003	Wolfdrif, RSA; Clanwilliam, RSA, 2016	male (4), female (4)
*Karoophasma botterkloofense* Klass, Picker, Damgaard, van Noort & Tojo, 2003	Calvinia, RSA, 2017	male (1), female (1)
*Viridiphasma clanwilliamense* Eberhard, Picker & Klass, 2011	Clanwilliam, RSA, 2017	male (2), female (2)

## Scanning electron microscopy (SEM)

Tarsi of ethanol stored specimens were severed at the base of the tibia and dehydrated in an ascending ethanol series. Subsequently, samples were critical point dried (Leica EM CPD 300, Leica Microscopy GmbH), mounted on aluminum stubs and sputter-coated with 10 nm gold-palladium using an EM SCD500 sputter coater (Leica Camera, Wetzlar, Germany). The tarsi were observed using a TM3000 tabletop SEM (Hitachi High-Tech Corp., Tokyo, Japan) at 15 kV accelerating voltage. If necessary, the SEM Hitachi S4800 (Hitachi High-Technologies Corp., Tokio, Japan) was used at 5 kV acceleration voltage for higher magnifications. Overview images of the same tarsus from different directions were obtained by using a rotatable sample holder [25]. Images were cropped and aligned using Affinity Photo and Affinity Designer (Serif Ltd., United Kingdom). Distances (e.g. aspect ratios) were measured using ImageJ version 1.54d [26]. We used additional micrographs of *M. zephyra* from previous investigations to assess the tarsal morphology [6, 7] obtained from cryo-SEM using liquid nitrogen for the cryo-fixation of the freshly ablated tarsi as described therein.

## Body length measurements

To assess morphological differences between species in light of size differences across mantophasmatodeans, body length was used for comparison between species. We collected reported measurements from the literature [27–30] (see Supplementary Information S1) and measured the body length of all specimens examined in this study according to Klass et al. [28], i.e. from the anterior margin of the head to the posterior margin of the epiproct, using digital calipers (Alpha Tools Digital Caliper, Mannheim, Germany) to the nearest 0.01 mm.

## Terminology of micromorphological characters

Some micromorphological characters are named inconsistently in the literature. The small cuticular protuberances on the arolium are sometimes termed microtrichia (e.g [10]). and sometimes acanthae (e.g [6, 7]). for Mantophasmatodea. The same applies for the same character in Phasmatodea (see [21]). A similar situation is found for the hairs on the euplantulae. These are regarded as acanthae [6], hairs [10] or setae [7]. All of these terms originally relate to the cellular organization of the cuticle protuberances according to Richards & Richards [31]. Acanthae are defined as unicellular outgrowths, microtrichia are subcellular and setae consist of multiple cells. We consider the protusions on the arolium as acanthae and the hairs on the euplantulae as tenent setae according to their size, but further anatomical research is required to clarify the structural origin of these structures. As tenent setae is often used as a term for adhesive setae on insect tarsi we adopted this term for consistency. However, most of these tenent setae lack sockets typical for actual multicellular setae [2], hence, might more likely be acanthae.

## Results

### Overall tarsal morphology

**Fig. 1:**
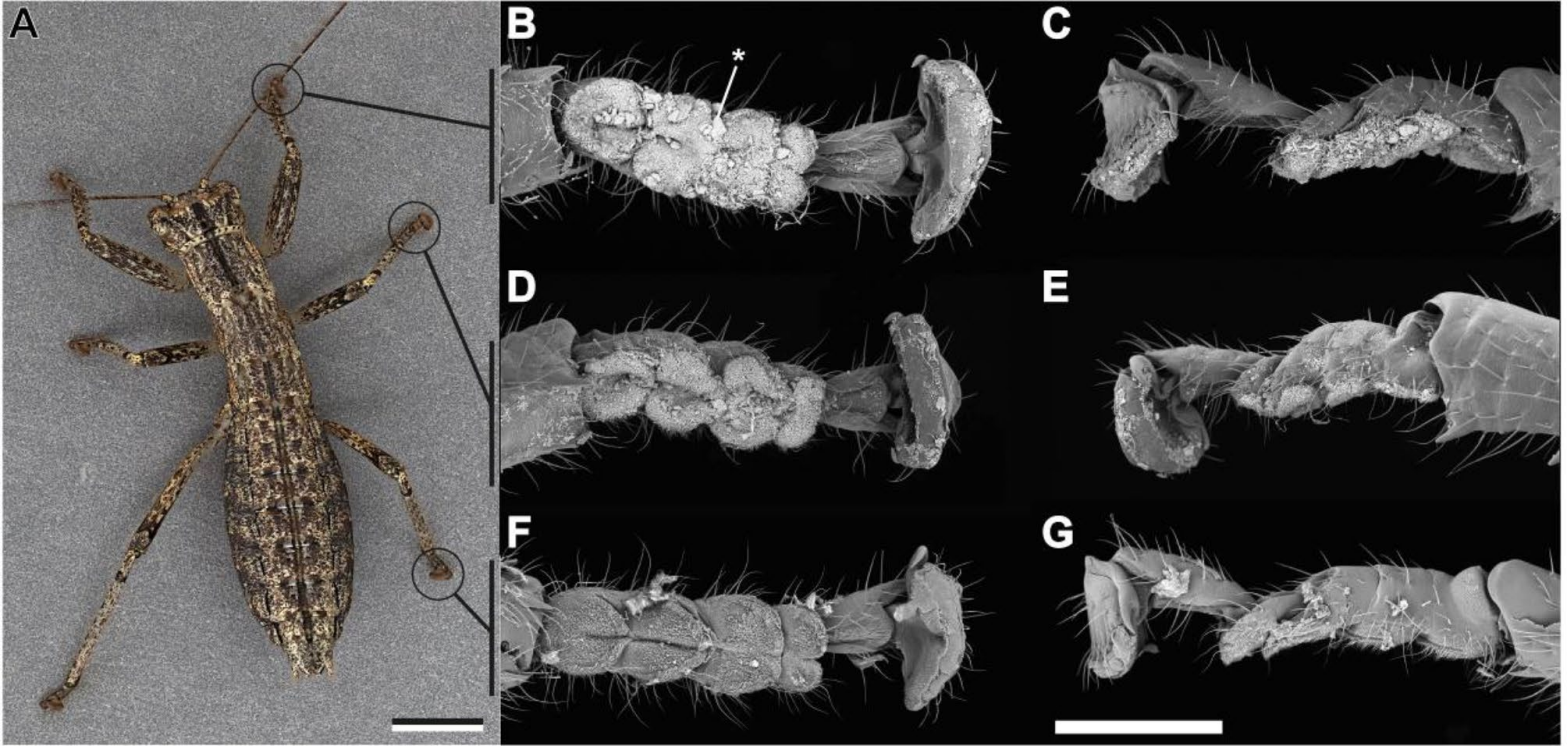
Tarsi of *Karoophasma biedouwense*. **A**. Adult female, image by S. Küpper. **B-G**. Overview SEM images of the female tarsal morphology. **B**, **D**, **F.** ventral views. **C**,**E**, **G**. lateral views, ventral side facing downwards. **B**, **C**. Protarsus. **D**, **E**. Mesotarsus. **F**, **G**. Metatarsus. The setae of the euplantulae are largely covered by soil particles (asterisk), especially on the protarsus (**B**) and mesotarsus (**D**). Scale bars = **A** 1 mm; **B**–**G** 500 μm

**Fig. 2:**
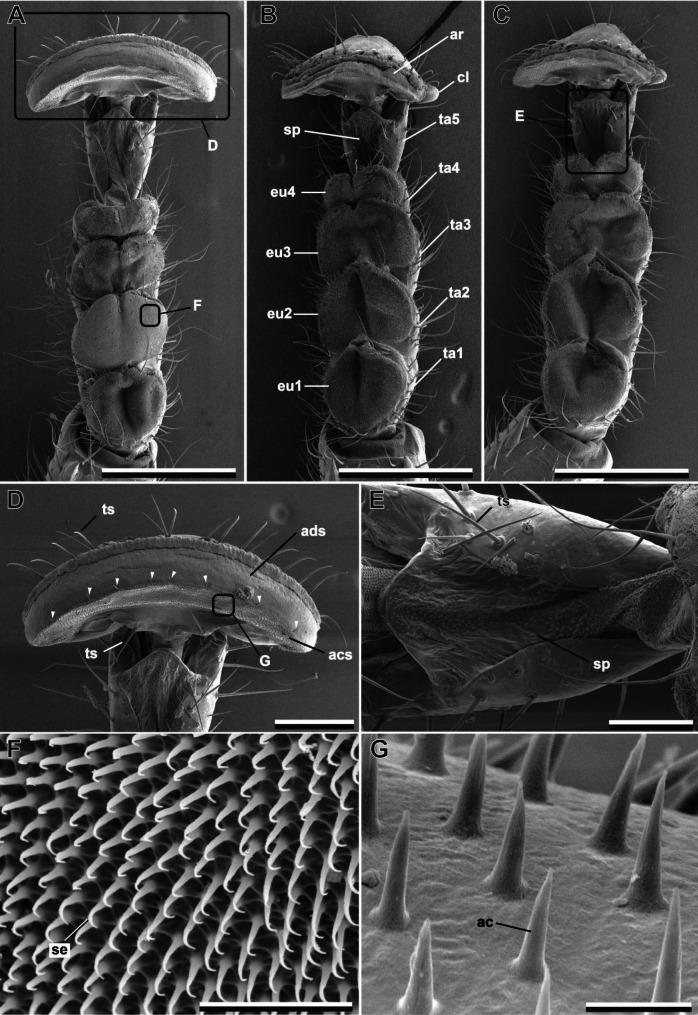
Overview of the tarsal morphology of *Austrophasma gansbaaiense*, female. **(A)** Protarsus. **(B)** Mesotarsus. **(C)** Metatarsus. **(D)** Arolium. **(E)** Tarsomere 5. **(F)** Adhesive setae of the euplantulae. **(G)** Acanthae on the arolium. ac, acantha; acs, acanthae zone; ads, adhesive zone; ar, arolium; cl, claw; eu1–4, euplantula 1–4; ta1–5, tarsomere 1–5; se, adhesive seta; sp, soft membranous pad; ts, trichoid sensilla; arrowheads, trichoid sensilla line between acs and ads. Scale bars = **A**–**C**, 500 μm; **D**, 200 μm; **E**, 100 μm; **F**, 20 μm; **G**, 5 μm

The tarsi of the three leg pairs (i.e. fore legs, middle legs and hind legs) are homogenous within the same individual (Fig. 1). There are no noticeable differences in the overall morphology of the tarsi. Therefore, with few exceptions, not all three leg pairs’ tarsi are shown here for the majority of the investigated species. All tarsi consist of five tarsomeres, of which the proximal three are somewhat fused, but can be distinguished by dorsal grooves (Fig. 1). The proximal four tarsomeres bear hairy euplantulae (Fig. 2A–C). The fifth tarsomere (ta5) bears a membranous pad ventrally, that is partially covered by acanthae, i.e. unicellular cuticular outgrowths according to Richards & Richards [31]. One arolium and two claws are located on the pretarsus (Fig. 2). Euplantulae, arolia, and the membranous area on ta5 consist of soft cuticle, indicated by the deflation of these pads observed in most cases. These membranous areas are all surrounded by elongated trichoid sensilla (Fig. 2; ts).

## Arolia

**Fig. 3:**
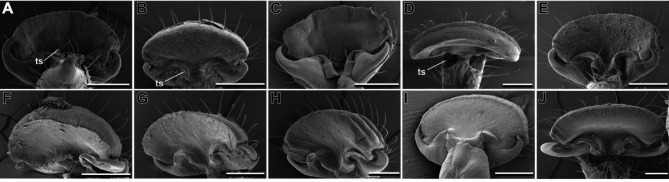
Comparative morphology of arolia. A. *K. biedouwense*, female protarus. **B**. *K. botterkloofense*, female protarsus. **C**. *H. montaguense*, male protarsus. **D**. *A. rawsonvillense*, female protarsus. **E**. *A. gansbaaiense*, female protarsus. **F**. *N. ookiepense*, female protarsus. **G**. *V. clanwilliamense*, male mesotarus. **H**. *S. paresisense*, male mesotarsus. **I**. *M. zephyra*, female metatarsus. **J**. *T. gladiator*, male mesotarsus. ts, trichoid sensilla. Scale bars = 200 μm

The mantophasmatodean arolium is a smooth attachment pad (sensu [2]). It does not bear elongated hairy attachment structures. Nevertheless, the ventral proximal area of the arolium is covered with acanthae in all species (Fig. 3). The area in which acanthae are found is distinctly separated by the smooth adhesive zone (Fig. 2D, arrowheads). A line of trichoid sensilla is separating these two areas. Two claws are present on every pretarsus. These claws are comparably small and do not protrude over the arolium. In contrast to most other insects, the claws are oriented to the proximal side of the arolium. In most species, two trichoid sensilla are situated at the base of the claws (Fig. 3, ts). These sensilla were not present in *S. paresisense* (Fig. 3H). On the macroscopic level, no other differences between the taxa were observed.

## Euplantulae

**Fig. 4:**
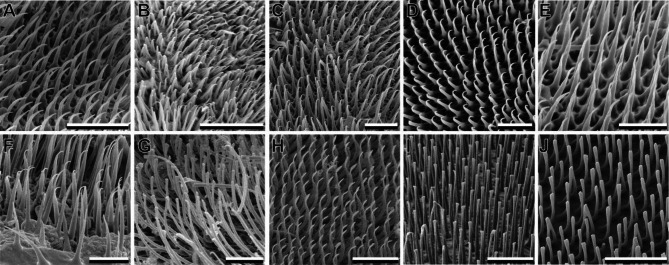
Comparative morphology of euplantula adhesive setae. **A**. *K. biedouwense*, female metatarsus. **B**. *K. botterkloofense*, male metatarsus. **C**. *H. montaguense*, male metatarsus. **D**. *A. rawsonvillense*, female mesotarsus. **E**. *A. gansbaaiense*, female protarsus. **F**. *N. ookiepense*, female protarsus. **G**. *V. clanwilliamense*, female mesotarsus. **H**. *S. paresisense*, male mesotarsus. **I**. *M. zephyra*, female metatarsus. **J**. *T. gladiator*, male metatarsus. Scale bars = **A**–**D**, **F**–**H**, 10 μm; **E**, **I**, **J**, 5 μm

**Fig. 5:**
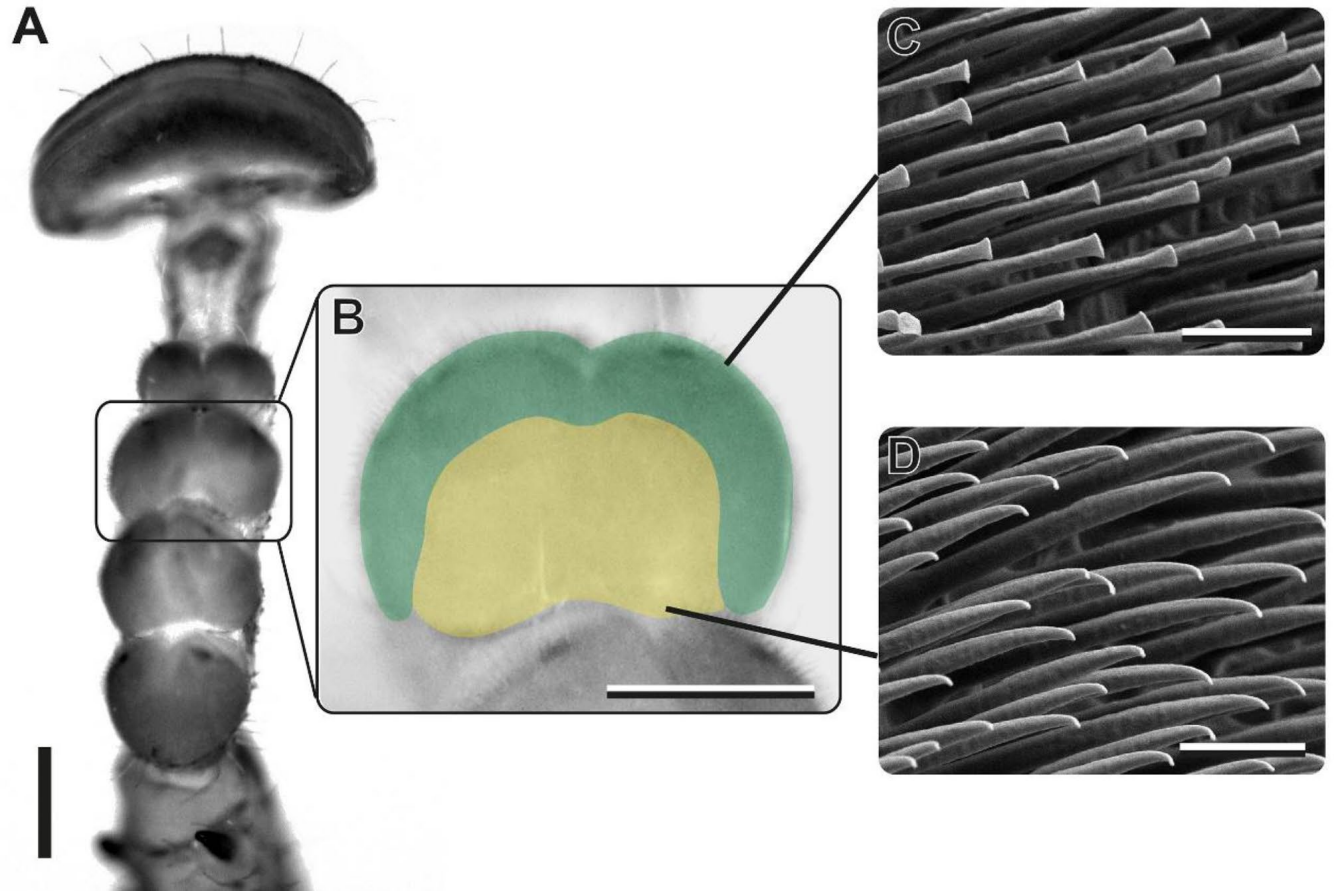
Distribution of spatulate setae in *M. zephyra*. **(A)** Light microscopy overview of the tarsus, ventral view. **(B)** Light microscopy image of the third euplantula. Colours indicate distribution of seta types. Green = spatulate setae, yellow = tipped setae. **(C)** Spatulate setae. **(D)** Tipped setae. Scale bars = **A**, 300 μm; **B**, 200 μm; **C**, **D**, 3 μm

The four tarsal euplantulae are large hairy attachment pads and cover the entire ventral face of the tarsomeres (Fig. 2A–C). No differences in the relative area of the tarsomere covered by the euplantulae were observed between the species. Every euplantula is densely covered by elongated tenent setae (Fig. 2F, se). The length of these setae varies within the same euplantula and increases from the center to the periphery of the pad (Figs. 1 and 2). Two different types of seta tips were observed within Mantophasmatodea. All species possess setae with single pointed tips (Fig. 4A–F), but setae with a terminal spatula were found in four species in addition to *M. kudubergense* [10]: *Viridiphasma clanwilliamense*, *S. paresisense*, *M. zephyra*, and *T. gladiator* (Fig. 4G–J). Interestingly, single tip setae are found in these species as well. Both types of setae co-occur on the same euplantula. While single tip (pointed) setae are situated in the central area of the attachment pad, spatulate setae are always found at the margin areas of the euplantulae in all species that possess this type of setae (Fig. 5).

### Fifth tarsomere

**Fig. 6:**
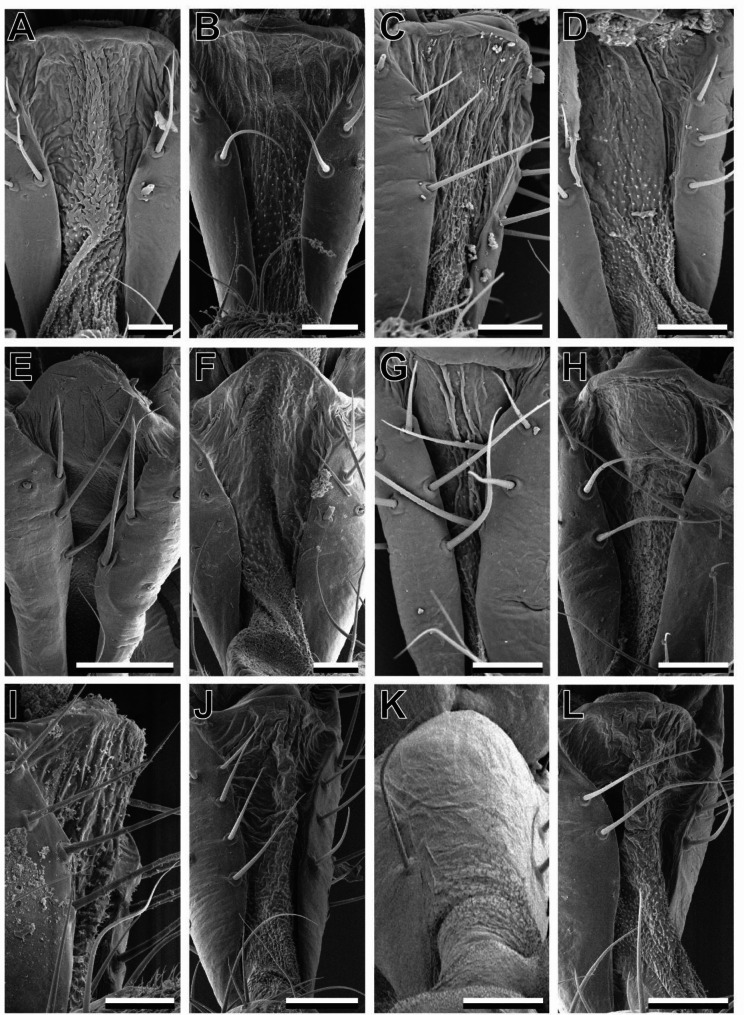
Comparative morphology of the fifth tarsomere. **A**, **B**. *K. biedouwense*, **(A)** male, **(B)** female. **C**, **D**. *K. botterkloofense*, **(C)** female, **(D)** male. **E**. *H. montaguense*, male. **F**. *A. rawsonvillense*, female. **G**. *A. gansbaaiense*, female. **H**. *N. ookiepense*, female. **I**. *V. clanwilliamense*, male. **J**. *S. paresisense*, male. **K**. *M. zephyra*, female. **L**. *T. gladiator*, male. Scale bars = 50 μm

The membranous ventral area of ta5 is present in all species and sexes which have been examined. It always projects from the intersegmental membrane between ta4 and ta5 to the distalmost tip of ta5 where it protrudes over the basis of the pretarsus (Fig. 1B–G). Shrinking artifacts of the cuticle suggest that it consists of soft cuticle and could be inflated (Fig. 6). All t5 membranous areas bear acanthae, which are short and in similar size across species. However, the distribution of these acanthae differs between species (Fig. 6) and, in one species (*K. biedouwense*) we observed sexual dimorphism of their distribution (Fig. 6A, B). All further species, of which both sexes were available had the same distribution of t5 acanthae in males and females. The posterior base of the membranous area was always covered with acanthae (Fig. 6). In total, we observed four different distribution patterns: base only (e.g. Figure 6B), median stripe (e.g. Figure 6A), shifted stripe (Fig. 6H) and full (Fig. 6F). In the base only distribution pattern, acanthae cover approximately the proximal half of the membranous area of ta5. This pattern is found in *H. montaguense*, *S. paresisense* and the females of *K. biedouwense*. The majority of species has a median stripe pattern. In this case, acanthae extend in the middle of the membranous pad distally towards the tip of the tarsomere. This distribution is found in *K. botterkloofense*,* A. gansbaaiense*,* V. clanwilliamense*,* M. kudubergense*,* M. zephyra*,* T. gladiator* and males of *K. biedouwense*. In *N. ookiepense* (shifted stripe) a similar, but comparably thinner, stripe of acanthae projects in distal direction shifted to the lateral side of the tarsus. The direction of this shift was always oriented to the posterior end of the animal, if the legs would have been stretched out orthogonal to the body. The acanthae coverage on ta5 of *A. rawsonvillense* (full, Fig. 6F) includes most areas of the pad up to the distal tip, except for the lateral sides.

### Acanthae on the arolium

**Fig. 7:**
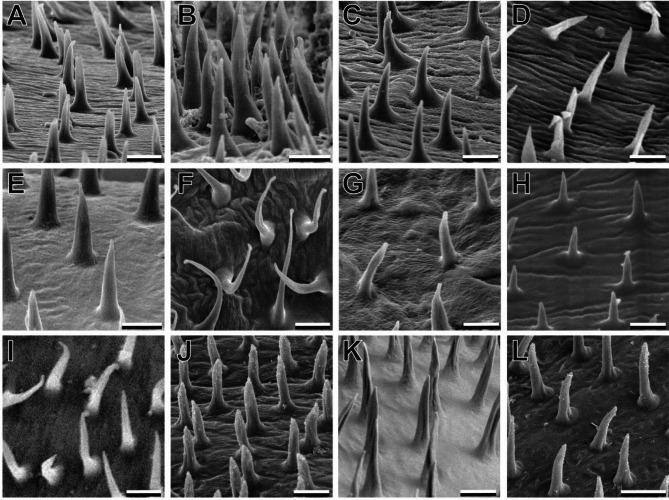
Comparative morphology of the arolium acanthae. **A**. *K. biedouwense*, female. **B**, **C**. *K. botterkloofense*, **(B)** female, **(C)** male. **D**. *H. montaguense*, male. **E**. *A. rawsonvillense*, female. **F**. *A. gansbaaiense*, female. **G**. *N. ookiepense*, female. **H**. *V. clanwilliamense*, male. **I**, **J**. *S. paresisense*, **(I)** female, **(J)** male. **K**. *M. zephyra*, female. **L**. *T. gladiator*, male. Scale bars = 3 μm

The acanthae found on arolium of different species covered the same areas, but differed in length (aspect ratio) and density across species (Fig. 7). While the base of a single acantha is mostly consistent, the length varies between species, resulting in different aspect ratios (width : length). We identified four different categories that were consistent within the same individual and in most cases between sexes. Two species with sexual dimorphism regarding acantha aspect ratio were observed: *K. botterkloofense* and *S. paresisense*. All aspect ratios ranged from 1:3 to 1:6. The shortest acanthae with aspect ratios of 1:3 are present in *V. clanwilliamense*, as well as in males of *K. botterkloofense*. Females of *K. botterkloofense* have acanthae with aspect ratios of 1:6. The only species with the same acantha aspect ratio was *A. gansbaaiense* (both sexes). The majority of species have acantha aspect ratios of 1:5, i.e. *H. montaguense*,* M. kudubergense*,* M. zephyra*,* T. gladiator* and females of *S. paresisense*. Males of the latter have an acantha aspect ratio of 1:4. The same aspect ratio was found in *N. ookiepense*,* A. rawsonvillense* and *K. biedouwense*.

Besides width-length relation the density of the acanthae differed across species, and (in two spp.) between sexes (Fig. 7). A full list of the acanthae aspect ratios, the distance between the acanthae and the density categories for all examined species and sexes is included in Table 2.

The distance between single acanthae slightly varied within the same specimen on the same arolium, we therefore assigned two categories for estimation of the acantha density. Minimum distances of 3 μm were considered narrow acanthae densities and minimum distances of 5 μm were considered wide (Table 2). Based on these categories all Austrophasmatidae had wide acanthae densities, except for in the male of *K. biedouwense* and the female of *K. botterkloofense* whose acanthae have a narrow acantha density. Narrow acantha densities were found in all other clades.

**Table 2:** Morphological measurements of the arolium acanthae and the body length. Categories (cat.) For density correspond to narrow = minimum distance 3 μm and wide = minimum distance 5 μm

species	sex	aspect ratio	distance (min-max) [µm]	density (cat.)	body length (min-max) [mm]
*K. biedouwense*	female	≤ 1:4	5–15	wide	13.0–19.4
* K. biedouwense*	male	≤ 1:4	3–6	narrow	11.3–15.6
* K. botterkloofense*	female	≤ 1:6	3–9	narrow	11.3–15.6
* K. botterkloofense*	male	≤ 1:3	5–10	wide	9.1–12.5
* H. montaguense*	male	≤ 1:5	5–10	wide	13.1
*A. rawsonvillense*	female	≤ 1:4	5–10	wide	9.1–21.1
* A. gansbaaiense*	female	≤ 1:6	5–15	wide	16.2–21.1
* A. gansbaaiense*	male	≤ 1:6	5–15	wide	13.1
*N. ookiepense*	female	≤ 1:4	5–10	wide	13.1–19.3
* V. clanwilliamense*	female	≤ 1:3	5–15	wide	14.0–16.0
* V. clanwilliamense*	male	≤ 1:3	5–10	wide	11.2–14.0
* S. paresisense*	female	≤ 1:5	3–6	narrow	16.8–20.5
* S. paresisense*	male	≤ 1:4	3–6	narrow	16.6–19.8
*M. kudubergense*	female	≤ 1:5	3–6	narrow	16.3–21.3
*M. zephyra*	female	≤ 1:5	3–6	narrow	19.7–23.6
*T. gladiator*	female	≤ 1:5	3–6	narrow	18.5
*T. gladiator*	male	≤ 1:5	3–6	narrow	26.0

## Discussion

### Functional morphology of the mantophasmatodean tarsus

Reasons for the characteristic uplifting of the arolium have been discussed in the literature [6, 7, 10], but were not subject to further experimental examination yet. Plausible reasons include (i) avoiding contamination or damage, (ii) saving tarsal adhesive secretion and (iii) achieving a trade-off between sufficient adhesion and support in emergency cases [10]. The strong contamination by soil particles observed on many euplantulae (Fig. 1) highlights the relevance of avoiding unnecessary contact with the ground to sustain its functionality. However, avoiding contamination could not be the main reason for the tarsal morphology of Mantophasmatodea, as most ground-dwelling insects that need to cope with contamination do not have similar attachment systems and usually lack adhesive pads that could be contaminated [22]. In contrast, the claws of mantophasmatodeans are unusually small for insects and likely do not engage much with the substrate. Claws often provide mechanical interlocking for attachment on mostly rough surfaces [32] and complement the function of the cuticular attachment pads [33]. For the use of the arolium in Mantophasmatodea, i.e. in emergency cases, such fast contact formation with the substrate, would probably not allow for reliable interlocking of the claws. Nevertheless, claws might still be required to provide structural support for the functionality of the arolium, as experiments on stick insects with ablated claws have shown that the attachment is also comparably reduced on smooth surfaces on which the arolia usually provide good attachment [34].

The surface of the arolium is separated into two areas, one with acanthae and one without (Fig. 2D). In other insects, arolia usually make contact with the substrate through a particular central area (see e.g [35–38]). This region corresponds to the smooth area of the mantophasmatodean arolium. Visualization of the real contact area of the arolium in Eberhard et al. [10] shows that this smooth area is brought into contact with the substrate, but only parts of the acanthae zone contact the substrate (Fig. 6 therein). While the membranous adhesive zone uses of wet adhesion (see e.g [5, 10]). yielding a large actual contact area, the acanthae only contact the substrate with their tips and actually reduce the potential real contact area. We hypothesize that this mechanism reduces the contact area, for example to avoid over-performance of the arolium and energy-loss due to more difficult detachment arising from the stronger adhesion. As the fast movement for contact formation of the arolium does interfere with a careful placement of the arolium, such a spacer system could balance a trade-off between good attachment and difficult release [10]. Alternatively, micro-patterned surfaces could allow for a two-options adhesive surface of which one is tuned for smooth surfaces and the other for rough ones, as on the tarsi of some orthopterans [39]. Similar low-aspect cuticle microstructures have shown a better performance of attachment pads with cuticular protrusions on rough substrates compared with smooth attachment pads [40–42]. If the acanthae on the arolium serve as adhesion-mitigating structures, the acanthae on ta5 might have the same purpose. Contamination and adhesion between parts of the same tarsus might be a risk for the functionality of the pretarsus. The dissimilar distribution of the acanthae across the species might be a result of differences in the posture of the pretarsus and distal tarsomere. Furthermore, the membranous area on the ventral side of ta5 might also work as a supplementary attachment pad, like it is found in other insects [21, 43–45].

**Fig. 8:**
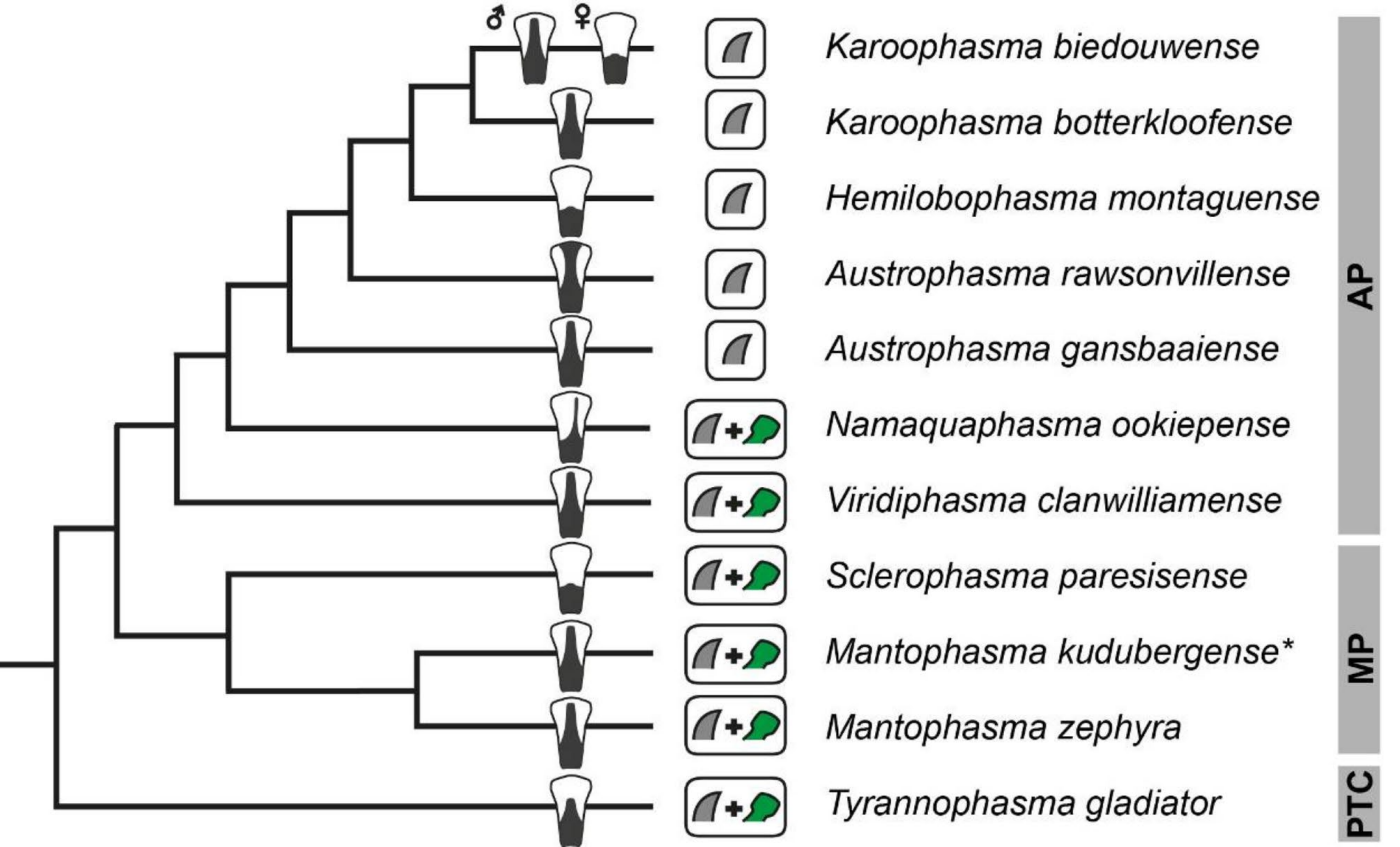
Summary of morphological disparity of the tarsal attachment system across Mantophasmatodea. Consensus cladogramm simplified from Eberhard & Picker [23] and Buder & Klass [55]. Pictograms show character states of euplantula setal tip morphology (gray, pointed tip; green, spatula) and distribution of acanthae on tarsomere 5 (terminal branches). Asterisk = data obtained from Eberhard et al. [10]. AP, Austrophasmatidae; MP, Mantophasmatidae; PTC, *Praedatophasma/Tyrannophasma*-clade

**Fig. 9:**
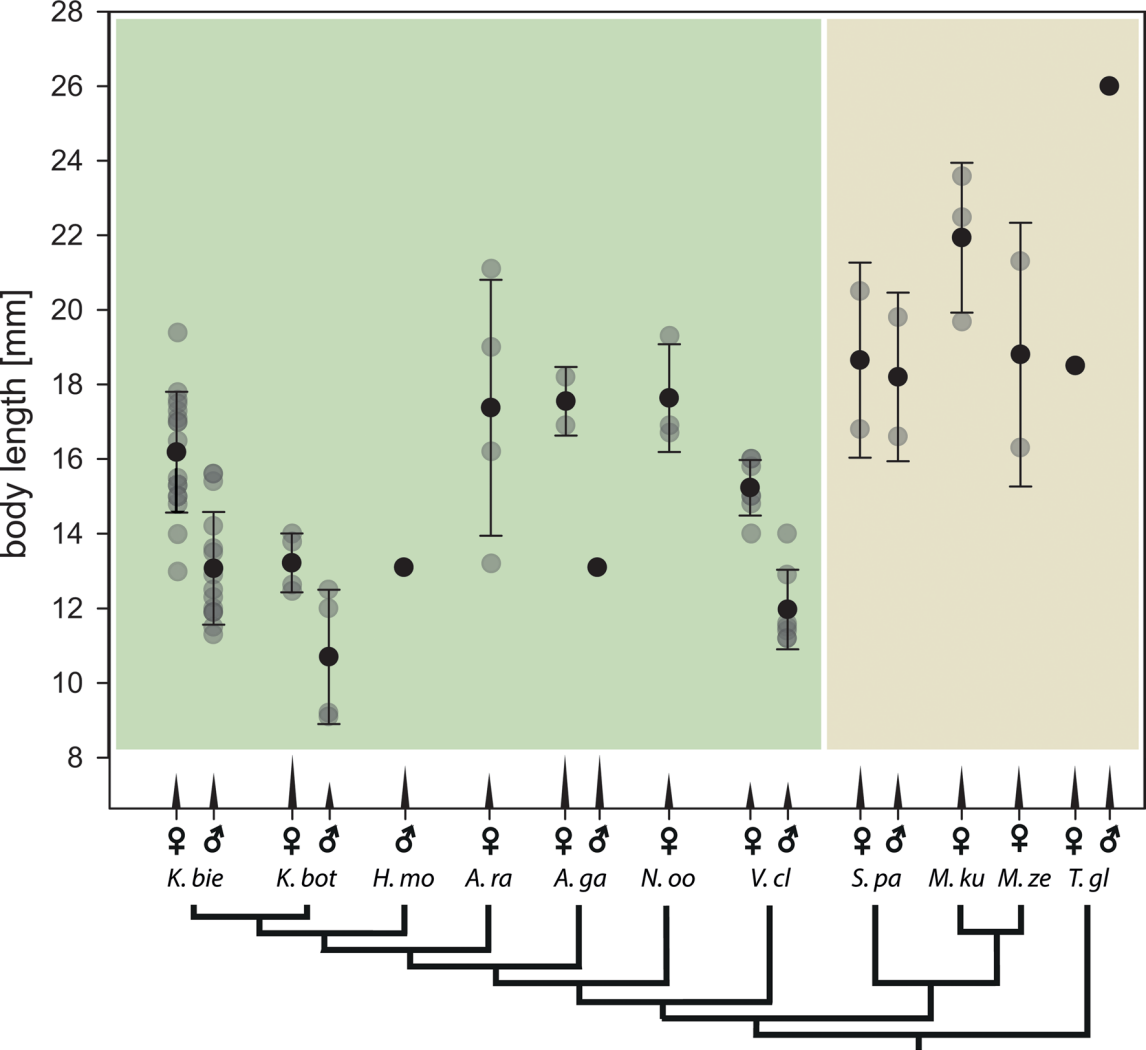
Summary of acanthae morphology across Mantophasmatodea in relation to the body length. The cladogram on the x-axis equals to the cladogram in Fig. 8. X-axis ticks represent aspect ratios (width : length) of the arolium acanthae in the respective sex. Coloured background indicates acanthae density (green = widely spaced, yellow = narrow). Sexual dimorphisms in acanthae density were omitted

The combination of smooth arolia and hairy euplantulae potentially results from demands that favor either of both systems. The fast movement and sudden impact of the arolium could require an attachment pad that does not consist of loose fibrils [10]. The hairy euplantulae in contrast might have other benefits for attachment during locomotion. While hairy attachment pads could be beneficial in the typical habitats of mantophasmatodeans, if they cope with the particulate contaminations in such arid environments [14], the actual influence of contaminations and respective efficiency of the different self-cleaning mechanisms between smooth and hairy attachment systems in insects is ambiguously addressed in experiments (e.g [46–49]). While substrate contact of the arolia is avoided most of the time, the setae on the euplantulae are permanently in contact with the ground. One factor influencing susceptibility of hairy attachment pads to contamination could be the morphology of the tip of the setae. There is a difference in the setal morphology across Mantophasmatodea (Fig. 8). To date, we cannot connect the apparent loss of spatulae within Austrophasmatidae to clear patterns of habitat preferences or other life history traits, as such are not known for these taxa. Instead, the size of mantophasmatodeans follows a similar trend: species with spatulate setae are mostly larger, while species without tend to be smaller (Fig. 9). Spatulae in principle increase the real contact area for adhesion due to their softness [3, 5, 50, 51] and, hence, spatulate setae generate stronger attachment compared to setae with a single tip. The spatulae are exclusively present in the peripheral areas of the euplantulae (Fig. 5) in which the tenent setae are strongly curved (Fig. 4) contributing to an expansion of the area available for adhesion compared to the area of the euplantula itself. Larger animals require disproportionally larger real contact area [52–54]. Consequently, the larger size of the species possessing spatulate setae could explain their presence in these taxa.

### Ground pattern of the mantophasmatodean attachment system

The overall tarsal morphology was similar in all species and corroborates the main pattern described already [13, 55]. However, spatulate setae [6, 7, 55] are not present in all species, but are lacking in most Austrophasmatidae (Fig. 8). Although the legs are partially used for different purposes, i.e. only fore legs are used for catching prey, the tarsal morphology does not differ across leg pairs.

Grylloblattodea (ice crawlers), the sister group of Mantophasmatodea [9], likely share only symplesiomorphic traits with heelwalkers in regard to their tarsal morphology. The presence of five tarsomeres, two pretarsal claws and euplantulae on the tarsomeres, although of different structure, is shared between Xenonomia (Grylloblattodea + Mantophasmatodea) and Phasmatodea [6, 7, 20, 21]. Eukinolabia, i.e. Phasmatodea (stick and leaf insects) + Embioptera (web spinners), is the sister group to Xenonomia [9, 56, 57]. However, embiopterans do not share most of these tarsal features with neither Xenonomia nor Phasmatodea [1, 21, 58], likely due to their specialized lifestyle and corresponding morphological adaptations [59–61]. The impact of the habitats and lifestyles likely has a stronger impact on the realization of certain attachment strategies than phylogenetic relatedness [62]. The main differences between the tarsi of Grylloblattodea and Mantophasmatodea is the missing arolium in Grylloblattodea and the morphology of the euplantulae, which are separated, small and smooth in Grylloblattodea [6], but large, fused and hairy in Mantophasmatodea (Fig. 2). Grylloblattodea tarsi interestingly possess an unpaired euplantula on tarsomere 5 [6, 13] instead of an arolium, that could be homologous to the membranous area on the same tarsomere in Mantophasmatodea. This euplantula likely replaces the arolium functionally in grylloblattids, but experimental studies on the arolia and euplantulae of stick insects [37, 63] have shown that the two pads differ in their mechanical properties and functional significance, with euplantulae being load sensitive friction pads and arolia being shear sensitive pads with strong adhesion. The importance of friction (attachment parallel to the substrate) might be consequently higher for Grylloblattodea compared to Mantophasmatodea. Despite being less closely related to Mantophasmatodea in comparison to Grylloblattodea, phasmids share more tarsal features with Mantophasmatodea. Phasmatodea includes species that bear smooth arolia and hairy euplantulae on the same tarsus [20, 21, 64]. Already within insects in general, hairy euplantulae evolved convergently in various lineages [1, 3, 5–7]. Interestingly, the tarsal morphology most similar to Mantophasmatodea is found in *Timema*, the basalmost split lineage within Phasmatodea: (i) the three proximal tarsomeres are fused [15], (ii) the arolium bears acanthae [6, 7, 39]. Nevertheless, these features likely indicate similar functional backgrounds, e.g. caused by the similar size and winglessness, rather than allow for interpretations as phylogenetic signal. Yet, both features occur solely in *Timema* and Mantophasmatodea, but not in any of the other closely related lineages. The specialized morphology of the arolium [10] is unique and consistently present in Mantophasmatodea.

### Inter-specific differences within Mantophasmatodea

There are notable differences between species in micromorphological features found on euplantulae and arolium (Figs. 4 and 7). Spatulate setae are present in all examined species of Mantophasmatidae, *Tyrannophasma gladiator* (the only species from the *Praedatophasma/Tyrannophasma*-clade) and *Viridiphasma clanwilliamense* (Fig. 8). They are absent in all remaining Austrophasmatidae examined herein. Functional considerations regarding the presence and absence of spatula are discussed below. Possible drivers for the occurrence of spatulae include particularly size and microhabitat of the organism [5].

In comparison, other lineages within Polyneoptera [18, 19, 21, 39], as well as in non-polyneopteran insect lineages [65–69] are more diverse in regard to their attachment systems. However, those lineages are represented by considerably more species and more diverse ecological backgrounds [62]. The most striking exception is Zoraptera, which includes a comparable number of species and a similarly uniform tarsal morphology [22]. In contrast to Mantophasmatodea, those insects do not have particularly specialized attachment systems, but instead lack dedicated adhesive organs. While it has been hypothesized for zorapterans that the lack of such organs conflicted with the diversification due to a lack of adaptiveness for settlement in diverse habitats, this scenario is unlikely for Mantophasmatodea. The complexity of the mantophasmatodean attachment system in contrast could be indicative for a strongly specialized use in a rather constant lifestyle [14].

The acanthae on the arolium of heelwalkers are a second feature that differs across species and is of functional relevance for the performance of the arolia [10], although the particular function is not elaborated in the literature (see below). Acanthae on the arolium are always pointy and differ primarily in their aspect ratios (i.e. the relationship between width and length) and density (Table 2). As these measures occasionally differed between the sexes, their role for the attachment performance might be involved in mating. The availability of material for investigation is limited for heelwalkers, as well as observations on the actual use of the attachment systems. As primarily ethanol stored material was examined and weight measurements are scarce for Mantophasmatodea we used body-length as a proxy for the body size (Fig. 9). The density of arolium acanthae is mostly overlapping with the major clades (Fig. 9), i.e. dense acanthae in Mantophasmatidae and the *Tyrannophasma/Praedatophasma*-clade and wider spacing in Austrophasmatidae (Table 2). However, the same transition is somewhat overlapping with differences in size between the species (Fig. 9) and different acantha densities could be related to size differences.

The distribution of ta5 acanthae (Fig. 8) shows no clear pattern within the mantophasmatodean phylogeny and could be a result of functional contexts (see below).

The attachment system shows a fairly constant morphology across species, likely because their ecology is so similar [70], and because the complementary use of fibrillary and smooth attachment pads probably copes with a large range of substrates. According to the extensively studied fossil record (e.g [27, 71–73] the attachment system appears to have remained rather uniform for more than 165 ma [73].

### Intraspecific differences

The density and aspect ratio of the acanthae on the arolium are partially subject to sexual dimorphism (Fig. 9). Although in general male heelwalkers are smaller than females, there is no clear correlation between size dimorphism and acantha density (Fig. 9). This difference in density might be related to the different body size of the two sexes, as the size mostly corresponds to the overall density of these acanthae (Fig. 9). The sexual dimorphism of the aspect ratio of the acanthae is somewhat ambiguous. The two species that display sexual dimorphismare not closely related, nor do they show a similar size dimorphism between the sexes (Fig. 9). The only pattern is that shorter acanthae are always found in the male (Table 2). The size dependence of the mantophasmatodean attachment system characters could be further investigated based on juveniles, as mantophasmatodeans are hemimetabolous and share a similar lifestyle and appearance between nymphs and adults [74].

Sexual dimorphsm might arise from different size, or from dissimilar selection pressures for the sexes [62]. This could be the case, if the sexes are adapted to different environmental conditions [75]. or due to mechanical reasons, if the attachment systems are used during copulation. There are various insect species, in which males possess dedicated structures to attach to the surface of females during mating (e.g [76–82]. The copula can take up to three days in Mantophasmatodea, in which the male does not feed, but the female retains mobility and continues to prey and feed despite the mounted male [14, 29, 83, 84]. Mating is one of the few occasions where the arolium was observed to be used [10, 14, 70] Females use their arolia mainly due to the higher weight of the copulating pair and the males to keep foothold on the females [10, 14, 70].

## Conclusion

The unique anatomy of mantophasmatodean attachment systems is strongly specialized and includes very few minor differences across species, notably in the presence of spatulae on the tenent setae of the tarsal attachment pads and the density of the acanthae on the pretarsal arolia. Both features potentially arise from overall size differences between the species. While it is possible that the specialization interferes with the adaptive potential of this system and results in uniform character sets across all mantophasmatodeans, it is likely that the versatility of the combination of the two different adhesive principles (smooth arolia and seta-based euplantulae) copes with the diversity of substrates the animals encounter.

## Electronic supplementary material

Below is the link to the electronic supplementary material.

Supplementary Material 1

## Data Availability

All corresponding data is accessible via the supplementary informations.
